# Metatranscriptomic Analysis of Virus Diversity in Urban Wild Birds with Paretic Disease

**DOI:** 10.1128/JVI.00606-20

**Published:** 2020-08-31

**Authors:** Wei-Shan Chang, John-Sebastian Eden, Jane Hall, Mang Shi, Karrie Rose, Edward C. Holmes

**Affiliations:** a Marie Bashir Institute for Infectious Diseases and Biosecurity, University of Sydney, Sydney, New South Wales, Australia; b School of Life and Environmental Sciences, University of Sydney, Sydney, New South Wales, Australia; c School of Medical Sciences, University of Sydney, Sydney, New South Wales, Australia; d Centre for Virus Research, Westmead Institute for Medical Research, Westmead, New South Wales, Australia; e Australian Registry of Wildlife Health, Taronga Conservation Society Australia, Mosman, New South Wales, Australia; f College of Public Health, Medical and Veterinary Sciences, James Cook University, Townsville, Queensland, Australia; University of Texas Southwestern Medical Center

**Keywords:** birds, evolution, metatranscriptomics, neurological syndrome, paresis, wildlife

## Abstract

Wild birds are major natural reservoirs and potential dispersers of a variety of infectious diseases. As such, it is important to determine the diversity of viruses they carry and use this information to help understand the potential risks of spillover to humans, domestic animals, and other wildlife. We investigated the potential viral causes of paresis in long-standing, but undiagnosed, disease syndromes in wild Australian birds. RNA from diseased birds was extracted and pooled based on tissue type, host species, and clinical manifestation for metagenomic sequencing. Using a bulk and unbiased metatranscriptomic approach, combined with clinical investigation and histopathology, we identified a number of novel viruses from the families *Astroviridae, Adenoviridae, Picornaviridae, Polyomaviridae, Paramyxoviridae, Parvoviridae*, and *Circoviridae* in common urban wild birds, including Australian magpies, magpie larks, pied currawongs, Australian ravens, and rainbow lorikeets. In each case, the presence of the virus was confirmed by reverse transcription (RT)-PCR. These data revealed a number of candidate viral pathogens that may contribute to coronary, skeletal muscle, vascular, and neuropathology in birds of the *Corvidae* and *Artamidae* families and neuropathology in members of the *Psittaculidae*. The existence of such a diverse virome in urban avian species highlights the importance and challenges in elucidating the etiology and ecology of wildlife pathogens in urban environments. This information will be increasingly important for managing disease risks and conducting surveillance for potential viral threats to wildlife, livestock, and human health.

**IMPORTANCE** Wildlife naturally harbor a diverse array of infectious microorganisms and can be a source of novel diseases in domestic animals and human populations. Using unbiased RNA sequencing, we identified highly diverse viruses in native birds from Australian urban environments presenting with paresis. This research included the clinical investigation and description of poorly understood recurring syndromes of unknown etiology: clenched claw syndrome and black and white bird disease. As well as identifying a range of potentially disease-causing viral pathogens, this study describes methods that can effectively and efficiently characterize emergent disease syndromes in free-ranging wildlife and promotes further surveillance for specific pathogens of potential conservation and zoonotic concern.

## Abstract (precis)

Wildlife naturally harbor a diverse array of infectious microorganisms and can be a source of novel diseases in domestic animals and human populations. Using unbiased RNA sequencing, we identified highly diverse viruses in native birds from Australian urban environments presenting with paresis. This research included the clinical investigation and description of poorly understood recurring syndromes of unknown etiology: clenched claw syndrome and black and white bird disease. As well as identifying a range of potentially disease-causing viral pathogens, this study describes methods that can effectively and efficiently characterize emergent disease syndromes in free-ranging wildlife and promotes further surveillance for specific pathogens of potential conservation and zoonotic concern.

## INTRODUCTION

Emerging and reemerging infectious diseases in humans often originate in wildlife, with free-living birds representing major natural reservoirs and potential dispersers of a variety of zoonotic pathogens (1). Neurological syndromes, such as paresis, are of particular concern, as many zoonotic viral pathogens carried by wild birds with the potential to cause neurological disease are also potentially hazardous to poultry, other livestock, and humans. Examples of this phenomenon include Newcastle disease virus (NDV, avulavirus 1; family *Paramyxoviridae*), West Nile virus (WNV, family *Flaviviridae*), and avian influenza viruses (family *Orthomyxoviridae*) (2). Importantly, with growing urban encroachment, the habitats of humans, domestic animals, and wildlife increasingly overlap. A major issue for the prevention and control of wildlife and zoonotic diseases is how rapidly and accurately we can identify a pathogen, determine its origin, and institute biosecurity measures to limit cross-species transmission and onward spread. With these ever-changing environments, wildlife are also at risk from a conservation perspective, and a number of emerging viral pathogens (WNV, Usutu virus, avian poxvirus, avian influenza virus, Bellinger River snapping turtle nidovirus) have had adverse population-level impacts (3–8). A fuller understanding of the diversity of the viral community and the ecology of microbes associated with urban wildlife mass mortality and emergent disease syndromes will improve our capacity to detect pathogens of concern and improve conservation and public health interventions (3).

Metatranscriptomic approaches (i.e., total RNA sequencing) have revolutionized the field of virus discovery, transforming our understanding of the natural virome in vertebrates and invertebrates (9, 10). This method relies on the unbiased sequencing of non-rRNA and has been used to identify novel viral species in seemingly healthy animals. In Australia, metatranscriptomic approaches have been used with invasive cane toads (Rhinella marina) (11), waterfowl (12, 13), fish (14), and Tasmanian devils (Sarcophilus harrisii) (15). These approaches have also been applied diagnostically, including in domestic animals such as cats (Felis sylvestris) (16), dogs (Canis lupus familiaris) (17), cattle (Bos taurus) with respiratory diseases (18–20), and pythons (*Pythonidae*) with neurological signs (21). Notably, metatranscriptomics has also been used to identify bacterial diseases, such as tularemia in Australian ring-tailed possums (Pseudocheirus peregrinus) (22). Hence, metagenomic approaches provide the capacity to comprehensively map microbial and viral communities, improving our understanding of animal health and zoonoses.

Investigations into wildlife diseases are often neglected and under-resourced. Consequently, while many outbreaks and syndromes are reported, until recently, limited molecular screening has been performed to characterize the etiology where a novel organism is present. In Australia, several neglected and undiagnosed disease outbreaks have been described in wild avian species, including those of suspected viral etiology. Notable examples include two syndromes, termed “clenched claw disease” (23–28) and “black and white bird disease” (29–31), which affect rainbow lorikeets (Trichoglossus moluccanus) and several species of passerines, respectively.

Clenched claw syndrome (CCS) has been recognized as a form of paresis in rainbow lorikeets in eastern Australia, in which birds present recumbent with poor withdrawal reflexes and clenched feet. Although the syndrome may be multifactorial, a proportion of the cases are characterized by nonsuppurative encephalomyelitis and ganglioneuritis, and these are suspected to have a viral etiology. Similarly, a series of morbidity and mortality events termed black and white bird disease (BWBD) have occurred in Australian magpies (Gymnorhina tibicen), pied currawongs (Strepera graculina), Australian ravens (Corvus coronoides), and magpie larks (Gallina cyanoleuca) along the Australian east coast (31). Diseased birds present in groups, either dead or paretic. Although these emergent disease syndromes are suspected to be caused by viral infections, they are poorly described, and to date no viral pathogens have been identified, such that the cause and mechanisms of disease remain elusive.

By exploiting metatranscriptomic approaches, validated with clinical manifestation and histopathological findings of infection, we investigated the potential viral etiology in historically archived outbreaks of birds fitting the syndrome descriptions associated with black and white bird disease and clenched claw syndrome in Australia, along with other sporadic cases where viral infection was suspected.

## RESULTS

### Clinical and histological description of rainbow lorikeets.

To formulate a syndrome description for CCS, pathology records from the Australian Registry of Wildlife Health from 451 rainbow lorikeets presenting between 1981 and 2019 were reviewed for unexplained nonsuppurative inflammation within the central nervous system. A total of 55 birds were found to match these search terms. The signalments, clinical signs, and histological lesions from these birds are summarized chronologically in Table S1 in the supplemental material. The index case was a juvenile female rainbow lorikeet found in Mosman, New South Wales (NSW), in November 1984, while the last known case was recorded in an adult female from the same location in May 2007. The majority of cases occurred in adult birds (28 adults, 20 juveniles, 7 age unspecified), including 24 males, 21 females, and 10 gender unspecified. Although cases were distributed throughout the year, twice as many CCS events occurred in October than any other single month.

**FIG 1:**
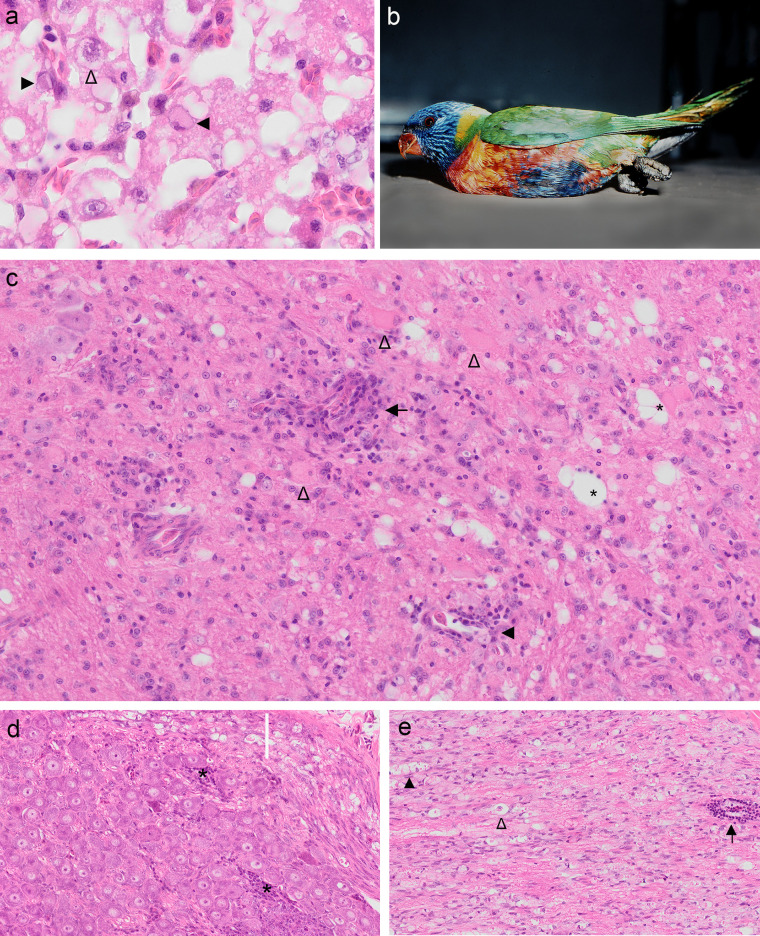
(a) Photomicrograph of rainbow lorikeet (case 4771.1) hepatocellular intranuclear inclusion bodies (filled arrowheads) and karyomegaly with a stellate chromatin pattern (open arrowhead). (b) Paretic rainbow lorikeet (case 4784.1) with clenched claws. (c to e) Photomicrographs of lesions characteristic of clenched claw syndrome in rainbow lorikeets (cases 5789.1, U1169, and U205). (c) Encephalitis within the central cerebellar white matter with perivascular lymphoplasmacytic infiltrates (black arrow), vascular intramural mononuclear cell infiltrates (filled arrowhead), gliosis, dilation of axonal chambers (*), and degenerating nerve cell bodies (open arrowheads). (d) Spinal ganglion with multifocal mononuclear cell infiltrates (*), swollen axons, and dilated axonal chambers within a white matter tract (white bar). (e) A peripheral nerve with perivascular mononuclear cell infiltration (black arrow) and Wallerian degeneration illustrated by dilated axonal chambers (filled arrowhead) and a macrophage within an axonal chamber signifying a digestion chamber (open arrowhead).

Clenched feet was the most prevalent presentation (*n* = 40), followed by an inability to fly (*n* = 13), unspecified neurological signs (*n* = 8), paresis (*n* = 6), leg paralysis (*n* = 3), wing paralysis (*n* = 2), head tilt (*n* = 3), tremors (*n* = 3), ascending or progressive neurological signs (*n* = 2), head bob (*n* = 2), opisthotonus (*n* = 1), ataxia (*n* = 1), and rolling (*n* = 1) (Fig. 1a and b). Body condition was noted in 29 records, and 11 birds were classified as being in good condition, 17 birds were considered thin or very thin, and one bird was emaciated. Less common presentations included flying into a window (*n* = 1) and predation (*n* = 2). The most common cause of death was euthanasia (*n* = 37).

Common microscopic lesions in CCS-affected lorikeets are illustrated in Fig. 1c to e and include mild to severe perivascular cellular infiltrates ranging from one to eight cells deep, composed of lymphocytes, plasma cells, and smaller numbers of macrophages. Gliosis, spongiotic change of the neuropil, Wallerian degeneration, and nerve cell body degeneration to necrosis were uncommon and restricted to those animals with moderate to severe inflammation. Nonsuppurative inflammation within the central nervous system was more prevalent and more severe within the caudal brainstem (28/54), cerebellum (30/54), and spinal cord (44/49) than in the cerebrum and anterior brain stem (12/54). Peripheral nervous system changes were also noteworthy, as 5 of 47 birds examined had nonsuppurative spinal ganglioneuritis, and 18 of 19 birds examined had nonsuppurative neuritis, often with Wallerian degeneration (13/19). An adult, male red-collared lorikeet (Trichoglossus rubritorquis) from Queensland (QLD) was also found with a history of euthanasia following presentation with clenched feet, ataxia, and thin body condition. Histological changes in this animal were consistent with CCS, including nonsuppurative lesions that were mild in the cerebrum and more moderate in the brain stem, cerebellum, and spinal cord.

Liver tissues from an adult, male rainbow lorikeet with histological findings of moderate hepatocellular single-cell necrosis, karyomegaly, and unusual amphophilic and variably shaped (stellate, discrete, and dense, and ground-glass) intranuclear inclusions (Fig. 1a) were included in the metatranscriptomic investigation based on suspicion of underlying viral infection. This bird presented recumbent, with extensive subcutaneous epithelial lined cysts encapsulating clusters of mites distributed along the wings and cranium and loss of the distal primary feathers and central tail feathers in a pattern suggestive of psittacine circovirus infection. We suggest likely viral coinfection since the tissue tropism and morphology of the hepatic inclusions were inconsistent with those associated with psittacine circovirus. However, we were able to exclude intoxication as a causative factor.

### Clinical and histological findings of black and white bird disease.

A total of 2,781 birds in the order Passeriformes were included in the analysis, which identified 51 birds with myocardial degeneration or nonsuppurative myocarditis. Two cases were excluded from the analysis due to incongruity of histological lesions compared with those of all other birds (*n* = 49) examined. The signalments, clinical signs, and histological lesions from these birds are summarized chronologically in Table S2.

**FIG 2:**
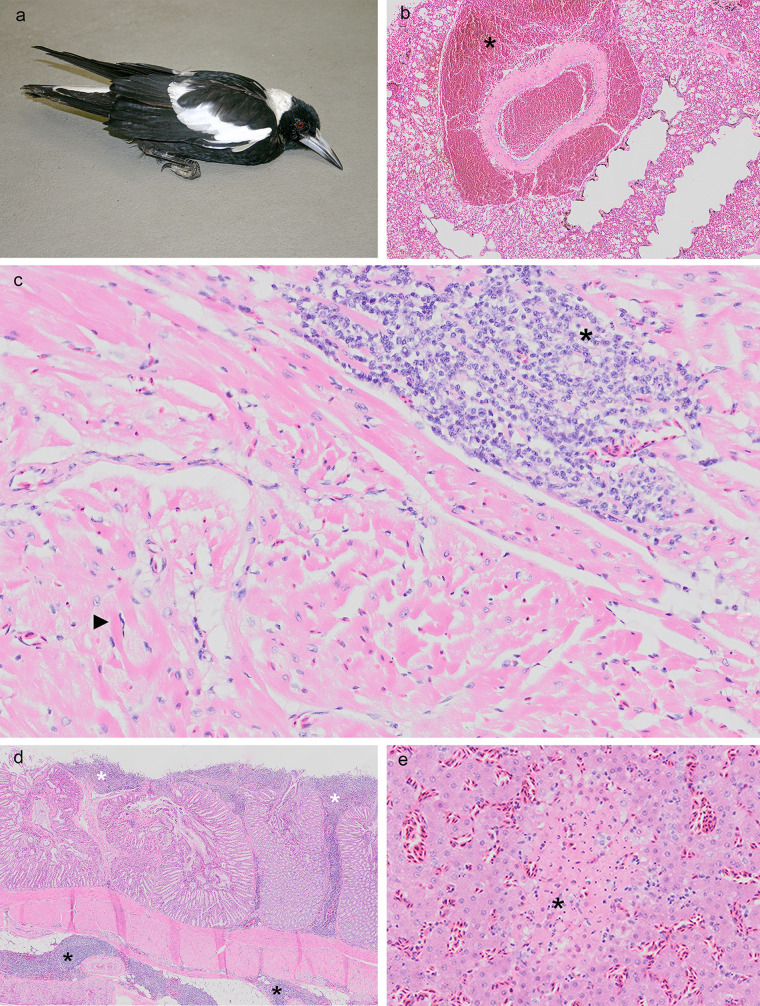
(a) A paretic, but alert, Australian magpie with black and white bird disease (case 5103.1). (b to e) Photomicrographs of lesions characteristic of black and white bird disease (case 10592.1 [b]; case 10592.2 [c to e]). Severe perivascular pulmonary hemorrhage (*). (c) Myocardium with a mononuclear cell infiltrate (*) and myocyte degeneration (black arrowhead) signified by hypereosinophilic myofibrils and a pyknotic and peripheralized nucleus. (d) Proventricular mononuclear cell infiltrates within the lamina propria (white asterisks) and surrounding serosal blood vessels (black asterisks). (e) Acute hepatic necrosis (*).

Although BWBD was first recognized during an epizootic extending across Victoria, NSW, and into QLD in 2006, records from the Australian Registry of Wildlife Health identified the index cases as magpies and currawongs found in Sydney and the NSW central coast in August 2003. The majority of birds examined were magpies (*n* = 26), currawongs (*n* = 15), and ravens (*n* = 6), with adults, juveniles, males, and females evenly represented. A solitary adult female figbird and one adult female magpie lark were also among the affected birds. Concurrent mortality was observed in crested pigeons (Ocyphaps olphotes), common koels (Eudynamys scolopacea), silver gulls (Larus novaehollandiae), and Indian mynahs (Acridotheres tristis). Presentation of affected birds varied, occurring as nine mass morbidity and mortality events and 25 sporadic cases. The birds examined from outbreaks in 2003, 2006, 2013, and 2015 represent a small fraction of affected birds and accumulatively total 105 animals reported formally and more than 250 reported informally. Suspected intoxication was a common history in those birds presenting en masse. Although seven birds died prior to veterinary examination, clinical signs among the remaining birds included paresis (Fig. 2a), recumbency or profound weakness (*n* = 27), loss of righting reflex or difficulty righting (*n* = 12), and, less commonly, dyspnea (*n* = 4), watery or bloody diarrhea (*n* = 4), clenched claws (*n* = 1), and a moribund state (*n* = 1). Although most birds were recumbent, they were not paralyzed, as they had good cloacal tone, withdrawal reflexes, and could stand and shuffle, flap, or walk across the room when stimulated. Most ravens had additional clinical signs indicative of central nervous system dysfunction, including ataxia, head tilt, circling, and unusual head posture (4/6). Other species were alert and could accurately grasp items with their beaks, despite being recumbent. The body condition of affected birds varied, with 21 in good body condition, while 27 were classified as thin, very thin, or emaciated.

Gross lesions in affected birds were rare, but included hydropericardium, epicardial hemorrhage, pulmonary congestion to hemorrhage, hemorrhage into the gastrointestinal lumen, and fibrinous serositis. Microscopic lesions characteristic of BWBD include the unifying feature of degeneration of cardiac myocytes with or without nonsuppurative perivascular and interstitial cardiac inflammation (Fig. 2b to e). Myocyte degeneration or nonsuppurative inflammation within skeletal muscle was observed in 24 of the 45 birds examined (four animals were unavailable for examination). Nonsuppurative inflammation of the central nervous system was observed in 25 birds. Lesions were most severe in the central cerebellar white matter and included one- to four-cell-deep perivascular cuffs, spongiotic change of the neuropil, degeneration to necrosis of nerve cell bodies, and multifocal gliosis. Other common histological features of the syndrome included acute hepatic necrosis or inflammation (25/49), necrosis or nonsuppurative gastrointestinal inflammation (21/49), nonsuppurative interstitial nephritis (8/49), mild vasculopathy to marked fibrino-necrotizing vasculitis (19/49), nonsuppurative perivascular infiltrates throughout serosal surfaces (20/49), and nonsuppurative interstitial pancreatitis (11/49). Less common lesions included perivascular pulmonary, cardiac and neural hemorrhage, meningitis, and peripheral neuritis. Ravens generally had more severe and widespread central nervous system lesions and lacked skeletal muscle, renal, and serosal inflammation that was evident in the other species. In birds other than ravens, paresis was most likely associated with lesions in striated muscle and the peripheral nervous system. Although leucocytozoons and microfilaria are common hemoparasites of the bird species examined here, and were found in earlier metagenomic data generated from these cases (32), these organisms did not accord with the lesions present. Ancillary diagnostic testing for known bacterial and viral pathogens, *Chlamydophila* species, and intoxication revealed no significant findings.

### Metatranscriptomic virus identification.

Archived samples, including brain, liver, heart, and kidneys from representative cases of diseased birds, were pooled and used to generate nine RNA-sequencing libraries that resulted in 7,725,034 to 26,555,569 paired reads per pool. From these data, we discovered eight viruses from the RNA virus families *Astroviridae*, *Paramyxoviridae*, and *Picornaviridae* and the DNA virus families *Adenoviridae*, *Circoviridae*, *Parvoviridae*, and *Polyomaviridae*.

### Avian avulavirus in brain samples of lorikeets with clenched claw syndrome.

The *Paramyxoviridae* are a large group of enveloped linear negative-sense RNA viruses that range from 15.2 to 15.9 kb in length. We identified paramyxovirus-like contigs in the libraries of the brain from rainbow lorikeets. A reverse transcription (RT)-PCR designed to amplify the paramyxovirus L protein (301 nucleotides [nt]) identified matching RNA in 4 of 5 brain tissues, corresponding to the four birds presenting with neurological symptoms. Ten overlapping RT-PCRs were then performed to confirm the genomic sequence (Table S4), revealing a typical 3′-N(455 amino acids [aa])-P(446 aa)-M(366 aa)-F(619 aa)-HN(528 aa)-L(2,263 aa)-5′ organization. Based on the phylogenetic analysis of the L and M proteins, the novel paramyxovirus identified here, termed avian avulavirus 5/Lorikeet/Australia/2016, fell into the clade comprising members of the genus *Avulavirus*, exhibiting 86.7% and 88.5% amino acid pairwise identity to its closest relative, avian paramyxovirus 5 (APMV-5)/budgerigar/Kuntachi/74 (GenBank accession no. NC_025361.1/YP_009094157).

**FIG 3:**
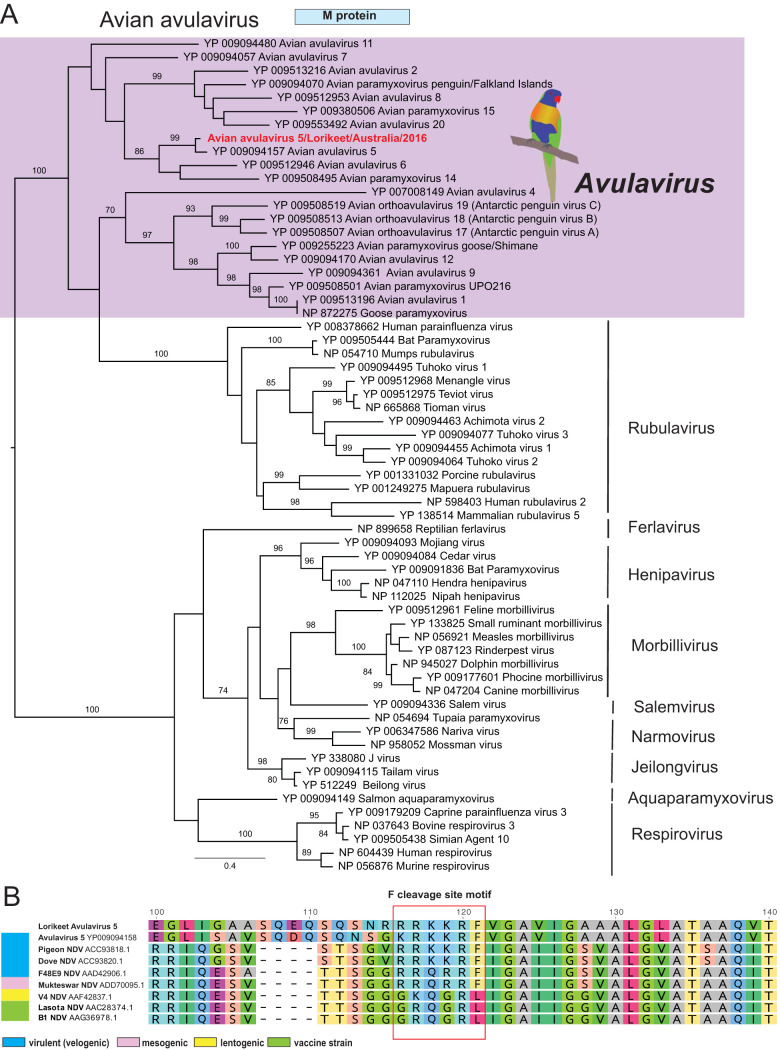
Phylogeny and characterization of a novel avian avulavirus identified from a rainbow lorikeet with clenched claw disease. (A) A maximum likelihood phylogeny of the M protein (366 amino acids) of avian avulavirus plus representative members of the *Paramyxoviridae*, including viruses from the genera *Avulavirus*, *Rubulavirus*, *Ferlavirus*, *Henipavirus*, *Morbillivirus*, *Aquaparamyxovirus*, and *Respirovirus*. The tree was midpoint rooted for clarity. The scale bar indicates the number of amino acid substitutions per site. Bootstrap values of >70% are shown for key nodes. (B) Characterization of the virulence determination site in the F protein by comparison to representative sequences of avulavirus 1. The typical RRKKR cleavage site motif is highlighted (red rectangle).

To provide a provisional assessment of the virulence of this novel virus, we compared F protein cleavage sites between our lorikeet avulavirus and known pathotypes of NDV (avulavirus 1) that commonly causes disease in avian species, including the velogenic, lentogenic, mesogenic, and asymptomatic vaccine types. The precursor F glycoprotein (F0) is cleaved into F1 and F2 subunits. The F protein cleavage position for virulent or mesogenic strains contain a furin recognition site comprising multiple basic amino acids. Notably, the lorikeet avulavirus had an F cleavage RRRKKRF motif identical to pathogenic NDV strains, suggesting its potential virulence, although this remains to be confirmed (Fig. 3).

### DNA viruses identified in sporadic cases or mortality events in rainbow lorikeets.

We identified three DNA viruses in the context of sporadic mortality events of rainbow lorikeets: avian chapparvovirus, beak and feather disease virus, and lorikeet adenovirus. In addition to the RNA-sequencing results, we extracted DNA from corresponding tissues for DNA virus validation through PCR and rolling-circle amplification (RCA) assays.

### Lorikeet chapparvovirus.

**FIG 4:**
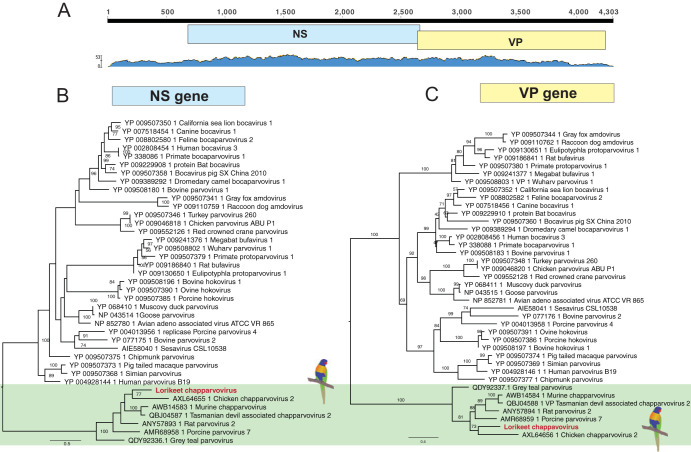
Characterization and phylogeny of the lorikeet chapparvovirus identified here. (A) Schematic representation and read abundance of the genome of lorikeet chapparvovirus. (B and C) Maximum likelihood phylogenies of the NS gene (B) and VP gene (C). The tree was midpoint rooted for clarity. The scale bar indicates the number of amino acid substitutions per site. Bootstrap values of >70% are shown for key nodes.

The *Parvoviridae* are a family of small, nonenveloped, dsDNA animal viruses with linear genomes of ∼5 kb in length. We identified parvovirus-like transcripts in both transcriptome sequencing (RNA-seq) libraries (RBL-L-2 and RBL-L-3) of the liver from diseased lorikeets (Table 1). Using RCA to enrich for circular DNAs, we recovered the complete genome of a novel lorikeet chapparvovirus, comprising 4,271 nt with two distinct open reading frames (ORFs) that encoded the nonstructural protein NS1 (670 aa) and the structural protein VP (542 aa). We designed specific primers to amplify the targeted VP region (∼248 nt) for screening both the RNA (positive in two liver samples) and DNA (all positive) products. In addition, we inferred two separate phylogenetic trees based on the complete NS1 and VP proteins to determine the evolutionary relationships between the lorikeet virus and other parvoviruses. These revealed that the closest relative to the novel lorikeet chapparvovirus identified here was an avian-associated red-crown crane parvovirus, yc-9 (GenBank accession no. KY312548.1), although this shares only 48.2% amino acid identity in NS1 and 46.9% identity in VP (Fig. 4).

**TABLE 1:** Details of the nine RNA-seq libraries, avian species, and disease syndromes studied here

Library	Host	Case no.	Organ(s)	Reads (pair reads)	Outcome or suspected disease^*a*^
RBL-L-2	Rainbow lorikeet	4771.2	Liver	26,555,569	Sporadic mortality events
RBL-L-3	Rainbow lorikeet	5604.1, 2989.1, 4575.1	Liver	25,509,669	CCS, sporadic mortality events
RBL-B-9	Rainbow lorikeet	8856.2, 5789.1, 5604.1, 2989.1, 4575.1	Brain	23,994,093	CCS, sporadic mortality events
RBL-B-46	Rainbow lorikeet	5604.1	Brain	24,868,929	CCS
Nowra-B-14	Australian magpie, magpie lark, pied currawong	9585.1, 9585.2, 9585.3, 9585.5, 9585.8, 9586.1	Brain	25,598,183	Nowra BWBD outbreak
Nowra-L-15	Australian magpie, magpie lark, pied currawong	9585.2, 9585.3, 9585.5, 9585.8, 9586.1	Liver	20,413,140	Nowra BWBD outbreak
BWBD-L-6	Australian magpie, pied currawong, Australian raven	5624.1, 5519.2, 7200.1, 6739.1	Liver	7,725,034	Archived BWBD
BWBD-B-8	Australian magpie, pied currawong, Australian raven	5624.1, 5519.1, 5519.2, 7139.1, 7200.1, 6739.1	Brain	25,779,043	Archived BWBD
BWBD-O-13	Australian magpie, pied currawong, Australian raven	5624.1, 5519.1, 7139.1, 7200.1, 6739.1	Heart, kidney	23,374,876	Archived BWBD

a CCS, clenched claw syndrome; BWBD, black and white bird disease.

### Beak and feather disease virus.

**FIG 5:**
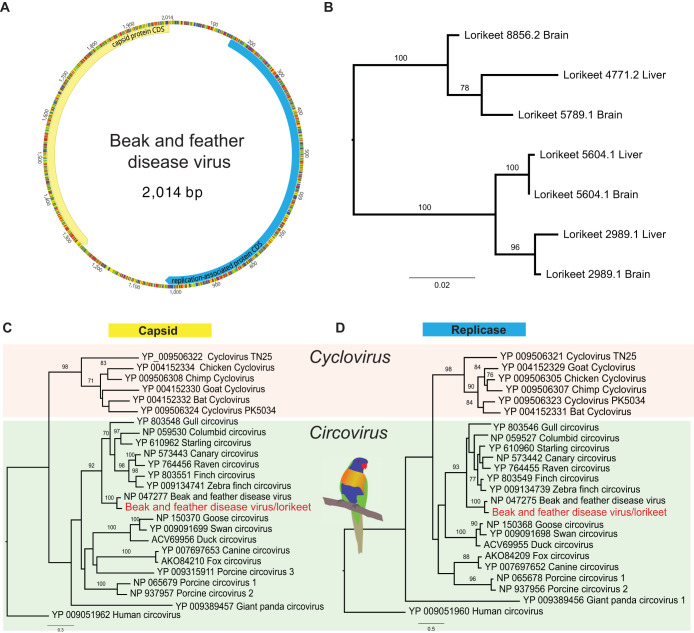
Genomic organization and phylogeny of the beak and feather disease virus (BFDV) identified in this study. (A) BFDV circular genome, annotated with two genes, encoding the capsid protein (Cap) and the replicase-associated protein (replicase). (B) Sanger sequencing of each case detected positive for BFDV. The ML phylogeny of the Cap gene (596 nt) was inferred, and the BFDVs identified from the same case fell into the corresponding clade. (C and D) Phylogenetic trees were estimated based on the Cap gene (C) and the replicase protein (D). The tree was midpoint rooted for clarity. The scale bar indicates the number of amino acid substitutions per site. Bootstrap values of >70% are shown for key nodes.

Beak and feather disease virus (BFDV), a member of *Circoviridae*, is highly prevalent in Australian wild birds, particularly psittacine species (33). We identified BFDV-like contigs in all lorikeet RNA libraries, sharing 95% nucleotide identity to known strains of BFDV. Remapping the raw reads to the closest BFDV strain (GenBank accession no. KM887928) gave coverage of the whole virus genome, comprising 2,014 nt and encoding a replication-associated protein (290 aa) and a capsid protein (247 aa) (Fig. 5A). We then screened all the RNA samples from rainbow lorikeets using PCR primers targeting the capsid protein (596 nt) (GenBank accession no. KM887928), with the PCR products then Sanger sequenced. The BFDVs identified from individual birds carried distinctive single nucleotide variants, and sequences from each case formed strong phylogenetic clusters, suggesting that these results are not due to cross-sample contamination (Fig. 5B).

### Lorikeet adenovirus.

**FIG 6:**
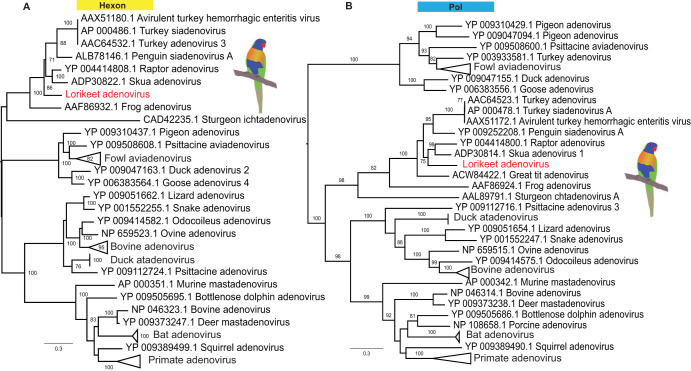
Phylogeny of the novel lorikeet adenovirus identified in this study. ML phylogenies of the hexon (A) and polymerase (B) proteins were inferred with representative members of the *Adenoviridae*. The tree was midpoint rooted for clarity. The scale bar indicates the number of amino acid substitutions per site. Bootstrap values of >70% are shown for key nodes.

Adenovirus-like contigs were identified from libraries of both the brain and liver of rainbow lorikeets. Phylogenetic analyses were performed based on the products of PCR primers designed based on the obtained sequences of polymerase (Pol, 843 aa) and hexon (580 aa) proteins. The virus identified, termed lorikeet adenovirus, exhibited 76% amino acid identity to the most closely related skua adenovirus (GenBank accession no. NC_016437.1) (Fig. 6).

### A novel lorikeet hepatovirus in lorikeets with clenched claw.

**FIG 7:**
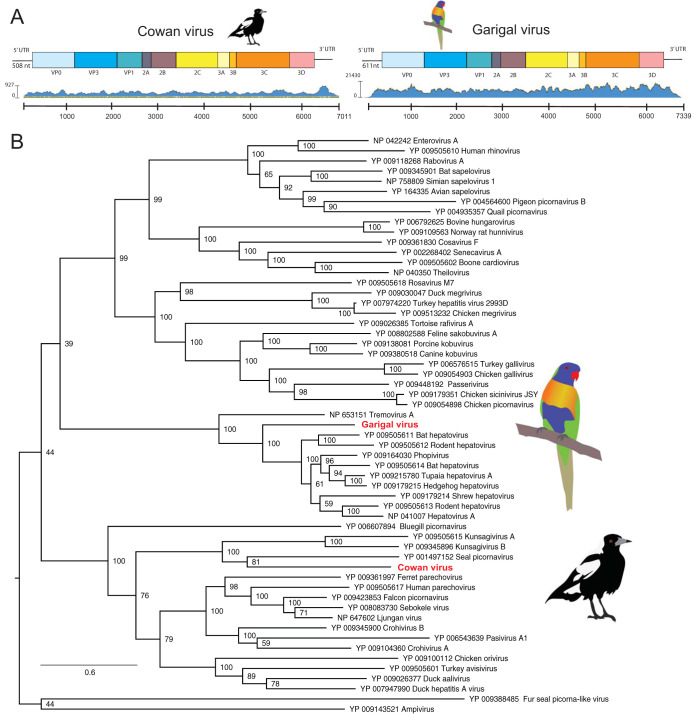
Cowan virus and Garigal virus identified in this study. (A) Schematic representation and read abundance of Cowan virus (upper left) and Garigal virus (upper right). (B) ML phylogenetic tree of the polyprotein (containing the RdRp gene) of both viruses. Bootstrap resampling (1,000 replications) was used to assess node support where it was >70%. GenBank accession numbers are given in the taxon labels. Trees were midpoint rooted for clarity. The scale bar indicates the number of amino acid substitutions per site.

A complete hepatovirus-like (*Picornavirales*) contig was identified in one of the libraries of the liver from the diseased lorikeets, exhibiting relatively high read abundance (∼10,000-fold coverage depth). The genome of this novel lorikeet hepatovirus, termed Garigal virus, is 7,339 nt in length and encodes a polyprotein of 2,070 amino acids, excluding poly(A) tails, that is similar in structure to most avihepatoviruses (Fig. 6). Interestingly, Garigal virus was most closely related to *Hepatovirus* sp. isolate HepV-bat3206/Hipposideros_armiger/2011 identified in a round-leaf bat, although these two viruses exhibit only 46.3% amino acid identity across the polyprotein. Recently, a lorikeet picornavirus, LoPV-1 (GenBank accession no. MK443503), was identified in fecal samples from rainbow lorikeets in China (34), although this exhibited only 17.4% amino acid identity to Garigal virus (Fig. 7).

### Novel picornavirus identified in black and white bird disease cases.

The *Picornaviridae* are a large family of single-strand positive-sense RNA viruses with genomes ranging from 6.7 to 10.1 kb in length. We identified a highly abundant complete picornavirus-like contig in the library of archived BWBD cases. Remapping raw reads to the picornavirus-like transcript showed complete genome coverage (∼300-fold depth). We named this novel black and white bird picornavirus Cowan virus. A RT-PCR targeting this picornaviral contig (∼220 bp) was positive in 2/6 of the brain tissues tested from an Australian magpie (Cracticus tibicen) and an Australian raven (Corvus coronoides). Notably, the two positive birds were those presenting with nonsuppurative encephalomyelitis.

Cowan virus exhibits classical picornavirus features, with a genome length of 7,011 nt excluding the poly(A) tails. This genome encodes a polyprotein of 2,070 amino acid residues, as well as a 5′ untranslated region (UTR) of 508 nt that includes the putative internal ribosome entry site (IRES). The genome organization of Cowan virus is similar to that of other picornaviruses, with the following characteristic gene order: 5′-VP4, VP2, VP3, VP1, 2A, 2B, 2C, 3A, 3B, 3Cpro, 3Dpol-3′ (Fig. 7). Cowan virus also exhibited such typical picornavirus features as rhv-like domains (aa 111 to 222 and aa 307 to 424), an RNA helicase (aa 1172 to 1271), and an RNA-dependent RNA polymerase (RdRp; aa 2903 to 2154). Phylogenetic analysis revealed that Cowan virus was most closely related to seal picornavirus (GenBank accession no. NC_009891.1), a member of the genus *Aquamavirus* (Fig. 7), although with very low amino acid similarity across the polyprotein: only 29.5% with Kunsagivirus A (GenBank accession no. YP_009505615.1) and 30.8% with seal picornavirus type 1 (GenBank accession no. YP_001497152.1). According to the International Committee on Taxonomy of Viruses (ICTV), members of a genus within the *Picornaviridae* should share at least 40% amino acid sequence identity in the polyprotein region. As such, Cowan virus may represent a new virus genus.

### A divergent black and white bird astrovirus in a BWBD outbreak from Nowra, NSW.

Several astrovirus-like reads were found in the RNA-seq library of the suspected BWBD (i.e., nonsuppurative encephalitis of undetermined etiology) outbreak from Nowra, NSW (Table 1). This novel virus, termed Nowra virus, was highly divergent in sequence and contained only 25 reads that covered the partial RdRp of astrovirus Pygoscelis/DT/2012 (GenBank accession no. KM587711.1) (see below).

**FIG 8:**
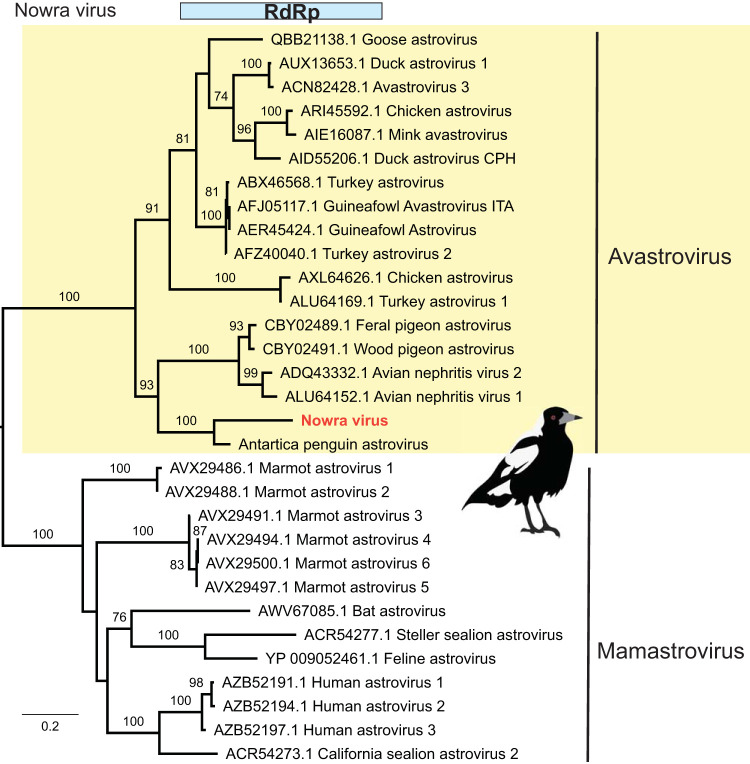
Phylogenetic analysis of Nowra virus. ML phylogeny of the partial RdRp protein (267 amino acids) of astroviruses was inferred along with representative members of the *Avastrovirus* and *Mamastrovirus* genera. GenBank accession numbers are given in the taxon labels. Trees were midpoint rooted for clarity only, and branch support was estimated using 1,000 bootstrap replicates, with those of >70% shown at the major nodes.

Reads from Nowra virus were used to develop PCR assays to bridge the gap in the RdRp. Using RT-PCR amplification of a 195-nt region targeting the RdRp, we obtained positive virus hits in 4 of 5 brain samples. Based on RdRp protein identity and phylogenetic analysis, there was 69.8% amino acid sequence similarity between Nowra virus and its closest relative, astrovirus Pygoscelis/DT/2012 (GenBank accession no. KM587711) sampled from Adelie penguins (Pygoscelis adeliae) in Antarctica (Fig. 8). Combining the PCR results with clinical and histopathology patterns, we suggest that Nowra virus might be the cause of the BWBD-suspected outbreak in Nowra (see Fig. 10B).

### Novel magpie polyomavirus in the Nowra outbreak.

**FIG 9:**
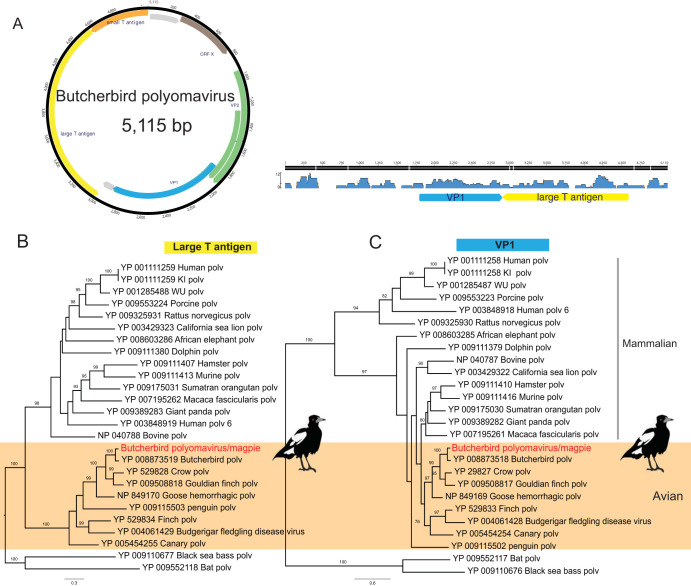
Genome characterization and phylogenetic analysis of the black and white bird polyomavirus identified in this study. (A) Schematic depiction and read abundance of the butcherbird polyomavirus/magpie. (B and C) ML phylogenetic trees of the large T antigen (blue) (B) and VP1 (yellow) protein (C) of the butcherbird polyomavirus/magpie. Bootstrap support (1,000 replicates) was used to assess node support where >70%. GenBank accession numbers are given in the taxon labels. The scale bars show the numbers of substitutions per site.

In addition to the novel astrovirus, our RNA-seq analysis identified a novel circular avian polyomavirus in brain and heart tissue from one nondiseased Australian magpie from the Nowra outbreak. Of note, the histopathology of BWBD in this case was inconsistent with polyomavirus infection, which is not known to be associated with myositis or encephalitis in birds. The genome of the novel magpie polyomavirus, termed magpolyV, comprised 5,115 bp with an overall GC content of 46.5%, and we were able to recover the full-length genome of this virus using RCA and PCR assays. Based on sequence similarity with existing reference polyomaviruses, we predict that the genome of magpolyV encodes capsid proteins VP1, VP2, and VP3 and open reading frame X (ORF-X) from the late region, as well as two alternative transcripts, large T antigen (LT) and small T antigen (ST) (Fig. 9). Consistent with most known polyomaviruses, magpolyV retains the typical conserved motifs and splicing sites in LT, including HPDKGG (DnaJ domain), LRELL and LLGLL (LXXLL-CR1 motif), LFCDE (LXCXE, a pRB1-binding motif). and GAVPEVNLE (ATPase motif). However, the consensus sequence CXCXXC for protein phosphatase 2A binding, mostly found in mammalian polyomaviruses, was absent. Phylogenetic analysis revealed that magpolyV consistently clustered within avian lineages, exhibiting 90.3% amino acid similarity to its closest relative, butcherbird polyomavirus isolate AWH19840 (GenBank accession no. KF360862), in the LT protein and 92% amino acid similarity in VP1.

## DISCUSSION

We characterized the clinical and histological features of CCS and BWBD and applied a metatranscriptomic approach to identify potential viral etiological agents in wild Australian birds presenting with hepatic inclusion bodies, paresis, and nonsuppurative myocarditis or encephalomyelitis. Through this combined approach, we were able to elucidate the complex nature of viral disease investigation amid multifactorial infections. In particular, metagenomic next-generation sequencing, in combination with traditional gross and histological examination, identified several candidate pathogens for disease syndromes that had been reported as undiagnosed for decades. Application of this methodology in the face of emergent disease syndromes or mass mortality events will facilitate the early detection of wildlife viruses, in turn contributing to disease control and wildlife conservation.

### CCS.

**FIG 10:**
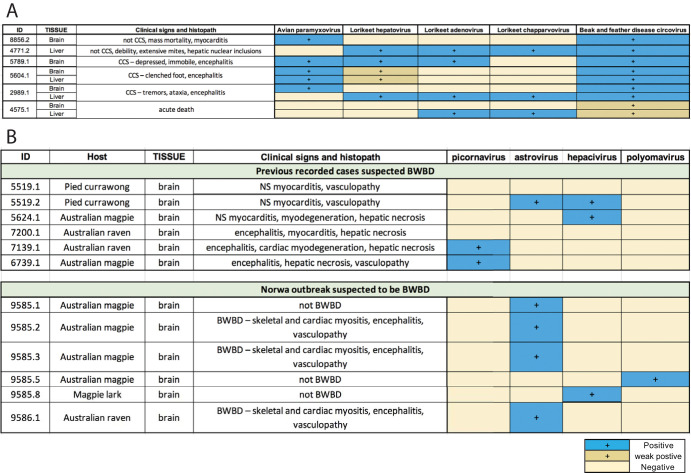
Summary of cases with metatranscriptomic pathogen identification and confirmation through PCR assays. (A) Clenched claw syndrome. (B) Black and white bird disease. Blue shading, PCR positive; yellow shading, PCR negative; dark yellow shading, PCR weakly positive; NS, nonsuppurative; CCS, clenched claw syndrome; BWBD, black and white bird disease.

A key element of our paper was the investigation of clenched claw syndrome (CCS), a paretic disease of unknown etiology in rainbow lorikeets (*Trichoglossus haematodus*) and a single red-collared lorikeet (*Trichoglossus rubritorquis*) in Australia. The diseased lorikeets presented with paresis and were often found recumbent or sitting on their hocks with feet clenched, the passive position for the avian foot. Affected birds kept in care progressively developed head tilt, ataxia, and paralysis. The disease syndrome has occurred primarily along coastal New South Wales and Southeast Queensland since the early 1980s. Although previous studies report 5% to 10% of free-living rainbow lorikeets rescued annually presenting with this syndrome, it seems likely that the clinical syndrome in fact encompasses two or more disease etiologies (35). For example, lead poisoning and plant-based intoxication have been proposed as contributing to syndromes described as clenched claw or drunken lorikeet syndrome (36). We focused on 55 cases of CCS characterized by nonsuppurative encephalomyelitis and often ganglioneuritis in which a viral etiology was suspected. The metatranscriptomic results, in conjunction with the clinical signs, histopathologic data, and confirmation through PCR assays, suggested that an avulavirus (APMV-5) potentially played a leading role in CCS pathogenesis in these diseased birds (Fig. 10A). An increasing incidence of clinical APMV infection in wild birds has been reported worldwide (37). APMVs have been categorized into 12 serotypes, with APMV-1 (NDV) having a significant economic impact on the poultry industry and population-level impacts on wild birds (37). Both NDV and APMV-5 have been associated with disease outbreaks in which mortality rates approach 100%, although the pathogenicity of APMV-5 varies substantially among aviary species. APMV-5 was first isolated from a fatal outbreak of caged budgerigars in Japan in 1974, followed by epizootic outbreaks in budgerigars in the United Kingdom (38) and Australia (39). Interestingly, APMV-5 does not replicate in chicken embryonated eggs, lacks a virion hemagglutinin, and is not pathogenic to chickens (40).

The gross and histological pathologies of avulaviruses vary depending on the viral pathotype and the susceptibility of the host. Although APMV-5 infection in budgerigars caused enteritis, APMV-3 in Australian *Neophema* species parrots has been associated with fatal neurological disease (41). The histological changes in rainbow lorikeets with CCS were most similar to neural lesions described for velogenic APMV-1 in poultry, free-ranging double crested cormorants (Phalacrocorax auritus), and pigeons (Columba livia domestica) (42–45). Collectively, Newcastle disease and CCS are characterized by gliosis, neuronal necrosis, nonsuppurative perivascular infiltrates, and white matter vacuolation. Notably, CCS-affected birds were devoid of the lesions in the respiratory and gastrointestinal tracts, lymphoid tissues, pancreas, liver, and kidney that are described for some APMVs and pigeon paramyxovirus (PPMV-1). Because of the possibility of cross-species transmission, the prevalence and pathogenicity of avian paramyxoviruses like APMV-5 circulating among wild birds in Australia clearly merit further investigation.

### Detection of avian chapparvovirus, avian adenovirus, and beak and feather disease viruses in rainbow lorikeets.

Chapparvoviruses are being increasingly identified in metagenomic studies and seemingly have a wide host range, including bats (46) and the feces of turkeys (47), red-crowned cranes (48), wild rats (49), and pigs (50). Importantly, a murine kidney parvovirus was recently found in immunodeficient laboratory mice with renal failure and kidney fibrosis (51), indicating that some chapparvoviruses result in highly virulent infections.

Avian polyomavirus, beak and feather disease virus, and avian adenovirus appear to be prevalent in psittacine species in Australia (52), compatible with the data presented here. As multiviral infection is common, our analysis of viral communities associated with neglected avian diseases enabled a comprehensive understanding beyond the one‐host, one‐virus system (13). Many known viral infections represent clinical issues in psittacine species due to their association with acute death, disease, the capacity to induce immunocompromise, and difficulties in treatment and control (53). These have been associated with a variety of RNA viruses, including avian paramyxovirus (37), avian influenza virus (54, 55), and avian bornavirus (56), and with DNA viruses, such as psittacine beak and feather disease virus (PBFD) (57), avian polyomavirus (APV) (58), psittacid herpesvirus (PsHV) (59), psittacine adenovirus (PsAdV) (60), poxviruses (61), and papillomaviruses (62).

### Role of astroviruses, polyomaviruses, and picornaviruses in black and white bird disease.

Viruses of the family *Astroviridae* infect multiple mammalian species, including humans, and also avian species and are most commonly associated with gastroenteritis and, rarely, neurological symptoms. A number of novel divergent astroviruses in wild birds have recently been discovered through metagenomic surveillance (63–65). Many avian astroviruses, including avian nephritis virus, duck and turkey astroviruses, and duck hepatitis virus 2, have considerable economic impact on the poultry industry, and these viruses are widely distributed in wild birds (66). Despite this, the diversity and ecology of these viruses, including their potential interspecies transmission events, are largely unknown. Previously, astrovirus-associated central nervous system impairment had been reported in mink, human, bovine, ovine, and swine (67–70).

In mammals such as dogs and cats, astroviruses are commonly isolated with enteric bacterial pathogens in sporadic gastroenteritis outbreaks (71), although encephalitis has been described in cattle and humans (72). In contrast, avian astrovirus infection manifests as a broader disease spectrum, including enteritis, hepatitis, and nephritis, but rarely neurological symptoms. The novel Nowra virus identified here was present in each bird with nonsuppurative encephalomyelitis, concurrent with myositis, coelomitis, and myocarditis from a single outbreak. This suite of histological lesions is unusual for avian astroviruses. A considerable proportion of BWBD-affected animals also had enteric and hepatic lesions similar to those described in astrovirus-infected poultry but also commonly associated with a variety of other viral pathogens. Further surveillance and *in situ* diagnostic modalities are required to reveal the association between Nowra virus, the clinical illness described, and the histological lesions observed.

A novel polyomavirus, magpolyV, was identified from the brain and heart of one of the nondiseased Australian magpies (*Cracticus tibicen*) that died in the same BWBD outbreak. An avian polyomavirus (APV), namely budgerigar fledgling disease polyomavirus, has been documented in young budgerigars and other psittacine species, causing feather abnormalities, abdominal distension, head tremors, and skin hemorrhages, reaching infection rates of up to 100% in aviaries worldwide (73). The clinical presentation and degree of susceptibility to APV infection range from skin diseases to acute death. In the case of nonpsittacine species, goose polyomavirus has been characterized as the etiologic agent of fatal hemorrhagic nephritis and enteritis of European geese (HNEG) (74). Other species of polyomaviruses have been identified in finches (75), crows (76), and butcherbirds (77), and a novel APV was recently associated with a fatal outbreak in canaries (78). Moreover, a recent study revealed an APV isolated from pigeon feces in China showing almost 99% identity with previously identified psittacine strains, suggesting a much broader host range of APVs and undetermined cross-species transmission (79).

Picornaviruses are commonly identified infecting a variety of avian species in metagenomic studies. In the context of overt disease, notable avian picornaviruses include avian encephalomyelitis viruses in chickens, pheasants, and turkeys (80), duck hepatitis A in ducklings (81), and a novel poecivirus that has been identified strongly associated with avian keratin disorder (AKD) in Alaskan birds (82). We identified two novel picornaviruses in this study, Garigal virus and Cowan virus, the pathogenic potentials of which merit further investigation. Interestingly, a novel lorikeet picornavirus (LoPv-1; GenBank accession no. MK443503) was identified in the feces of healthy rainbow lorikeets (*Trichoglossus haematodus*) in China (34), although the polyprotein shared only 12.6% similarity with the lorikeet hepatovirus identified here.

The high frequency of microbial coinfections suggests that rather than being the primary cause of the disease outbreak, some novel avian viruses might augment another pathogen, causing immunosuppression in immunocompromised hosts (83). Although we were successful in elucidating seven candidate viral pathogens that previously eluded detection, it is clear that additional experimental evidence such as *in vivo* inoculation experiments and *in situ* methodologies to identify pathogens within lesions are critical for clarifying the relationship between infection and the emergent disease syndromes we consider. Notably, the metagenomic approach utilized here is not limited to viruses and can be useful in the detection of bacteria, fungi, and protozoan and metazoan parasites (32). Previous studies have shown that the coinfection of pathogens like APMV-1 and avian influenza virus may greatly complicate disease outbreaks, for example, by modulating host immune responses, impacting the prevalence of other viral infections (84), and masking the presence of other pathogens (85). Thus, broader virome-scale studies such as that undertaken here provide a greater opportunity to understand virus-virus and virus-host interactions in more detail.

Due to the limited collection from natural outbreaks, more extensive sampling in the future will clearly help produce better case definitions and disease associations. Admittedly, the retrospective investigation may not have identified every potential case, particularly as viral infections may present differently in various avian species. Despite the relatively small number of disease cases sampled here, the utility of metatranscriptomics in the context of a thorough and multidisciplinary diagnostic approach is that it is able to identify viruses and other microbial species, including those that are highly divergent, with greater efficiency and scope than conventional diagnostic tests alone. The sequencing data and PCR assays developed here enhance diagnostic capabilities for avian disease and enable epidemiological surveillance to better understand the ecology and impacts of the viruses described. The investigation focused on identifying potential pathogens within specimens emanating from undiagnosed, but well-described clinical conditions in birds. These clinical syndromes and associated distinctive pathological changes have been previously described, enabling us to generate search terms to interrogate the archived collections. More broadly, our study highlights the value of proactive viral discovery in wildlife and targeted surveillance in response to emerging infectious disease events that might be associated with veterinary and public health.

## MATERIALS AND METHODS

### Animal ethics.

Wild birds were examined under the auspices of the NSW Office of Environment and Heritage Licenses to Rehabilitate Injured, Sick or Orphaned Protected Wildlife (no. MWL000100542). Diagnostic specimens from recently deceased birds were collected under the approval of the Taronga Animal Ethics Committee’s Opportunistic Sample Collection Program, pursuant to NSW Office of Environment and Heritage-issued scientific license no. SL10469 and SL100104.

### Sample collection.

Samples were collected between 2002 and 2013 from 18 birds, predominantly from within the Sydney basin and the coastal center of NSW. Fresh portions of brain, liver, heart, and kidney were collected aseptically and frozen at –80°C. Additionally, a range of tissues were fixed in 10% neutral buffered formalin, processed in ethanol, embedded with paraffin, sectioned, stained with hematoxylin and eosin, and mounted with a cover slip prior to examination by light microscopy. Giemsa stains were applied to a subset of tissue samples to determine whether protozoa were present within lesions.

### Historical case review.

The records of the Australian Registry of Wildlife Health dating back to 1981 were interrogated to identify rainbow lorikeets with clinical signs relating to the nervous system and those with unexplained nonsuppurative inflammation in the central nervous system. An additional search was conducted to identify passerine birds with unexplained nonsuppurative inflammation within cardiac or skeletal muscle. Retrieved records were investigated to determine the signalment, clinical signs, and histological lesions of affected animals.

The severity of nonsuppurative inflammation was graded on a scale of 0 to 4, where 0 indicated no discernible lesions and 4 represented severe and extensive mononuclear cell inflammation. Degeneration and necrosis were graded on a scale of 0 to 4, where 1 represented mild, multifocal single-cell degeneration or necrosis and 4 characterized extensive coagulative necrosis or malacia. Wallerian degeneration of the white matter tracts within the brain, spinal cord, and central and peripheral nerves was noted when present. In rainbow lorikeets, the lesions were graded in the cerebrum, brain stem, cerebellum, spinal cord, ganglia, and peripheral nerves. In the passerines, degeneration or necrosis and nonsuppurative inflammation were graded in skeletal and cardiac muscle, brain, liver, gastrointestinal tract, pancreas, blood vessel walls, and renal interstitium.

Avian influenza and NDV were excluded using real-time PCR on oropharyngeal, conjunctival, and cloacal swabs from 22 magpies and 12 other wild birds (including currawongs, magpies, and ravens). Serological testing of plasma from the same birds comprised hemagglutination inhibition to identify NDV antibodies and competitive ELISAs targeting avian influenza virus and flavivirus antibodies. Sixty-eight tissue samples from a subgroup of 13 magpies, currawongs, and ravens identified as having acute histological lesions suggestive of viral infection were subjected to further avian influenza virus-, NDV-, and WNV-specific RT-PCR and virus isolation. Virus isolation was attempted in both mammalian and insect cell lines (Peter Kirkland, personal communication.). Additional viral culture was attempted on the same samples inoculated into chicken embryos while assessing hemagglutinating agents and in Vero cells assessing cytopathic effect consistent with WNV infection. Clearview antigen capture ELISA (Unipath, Mountain View, CA) targeting *Chlamydophila* species was conducted on splenic tissue from eight birds with BWBD (magpies, currawongs, a raven, and a magpie lark). Liver and lung tissues from eight magpies and currawongs were subjected to routine aerobic and anaerobic bacterial and fungal culture. Finally, liver tissue from eight magpies and currawongs, representing the 2006 and 2015 BWBD epizootics, were subjected to toxicological testing to exclude the presence of a variety of acaricides, fungicides, organophosphates, carbamates, synthetic pyrethroids, and organochlorines and their metabolites.

### RNA extraction, library construction, and sequencing.

Viral RNA was extracted from the brain and liver, heart, and kidney samples of animals using the RNeasy Plus minikit (Qiagen, Germany). RNA concentration and integrity were determined using a NanoDrop spectrophotometer (Thermo) and TapeStation (Agilent). RNA samples were then pooled in equal proportions based on animal tissue type and syndrome. Illumina RNA libraries were prepared on the pooled samples following rRNA depletion using a RiboZero Gold kit (Epidemiology) at the Australian Genome Research Facility (AGRF), Melbourne, Australia. The rRNA depleted libraries were then sequenced on an Illumina HiSeq 2500 system (paired 100-nt reads).

### Virome metatranscriptomics.

Unbiased sequencing of RNA aliquots extracted from diseased animals in each outbreak were pooled based on host species, clinical syndrome, and histological findings as shown in Table 1. The RNA sequencing reads were trimmed of low-quality bases and any adapter sequences before *de novo* assembly using Trinity 2.1.1 (86). The assembled sequence contigs were annotated using both nucleotide and protein BLAST searches against the NCBI nonredundant sequence database. To identify low-abundance organisms, the sequence reads were also annotated directly using a BLASTX search against the NCBI RefSeq viral protein database using Diamond (87) with an E value cutoff of <10^−5^. Open reading frames were then predicted from the viral contigs in Geneious v11.1.2 (88), with gene annotation and functional predictions made against the Conserved Domain Database (CDD) (89). All sequences were aligned using the E-INS-i algorithm in MAFFT version 7 (90). Virus abundance was assessed using a read mapping approach in which reads were mapped to the assembled viral contigs using the BBmap program (91). Viruses were named in accordance with the ICTV guidance for each group in question.

### RCA assays for circular DNA viruses.

To recover the complete genomes of the polyomaviruses and circoviruses identified through our RNA-seq analysis, we enriched for circular DNA using rolling-circle amplification (RCA) (92). Briefly, genomic DNA was extracted from animal tissue and combined with a reaction buffer containing random primers, 1× phi29 buffer, dithiothreitol (DTT), bovine serum albumin, deoxynucleoside triphosphates (dNTPs), and phi29 DNA polymerase (Thermo Scientific, Australia) and then incubated at 30°C for 16 h. The RCA products were then purified using the Monarch PCR & DNA cleanup kit (NEB) and then quantified using the Qubit dsDNA broad-range assay. Nextera XT DNA libraries were then prepared and sequenced on an Illumina MiSeq to a depth of ∼2 million reads (2 × 150 nt).

### RT-PCR assays and Sanger sequencing.

Total liver and brain RNA from individual birds was reverse-transcribed with SuperScript IV VILO mastermix (Invitrogen). The cDNA generated from the sampled tissues was used for virus-specific PCRs targeting regions identified by RNA-seq. RT-PCR primers were designed to bridge any genomic gaps based on detected transcripts of the paramyxoviruses, polyomaviruses, adenoviruses, and astroviruses identified in the RNA-seq data (see Tables S3 and S4 in the supplemental material). Accordingly, all PCRs were performed using Platinum SuperFi DNA polymerase (Invitrogen) with a final concentration of 0.2 μM for both forward and reverse primers. All PCR products were visualized by agarose gel electrophoresis and Sanger sequencing. Long RT-PCR products were also sequenced using Nextera XT and the MiSeq platform, per the RCA assays described above.

### Phylogenetic analysis.

Conserved protein domains were used to determine the evolutionary relationships of the viruses identified here. In the case of RNA viruses, we used amino acid sequences of the RNA-dependent RNA polymerase (RdRp) that is the most conserved protein among this group. After removing all ambiguously aligned regions using TrimAl (93), phylogenetic trees were inferred using the maximum likelihood method (ML) implemented in PhyML version 3.0 (94), employing a Subtree Pruning and Regrafting topology searching algorithm and the LG model of amino acid substitution. Bootstrap resampling with 1,000 replications under the same substitution model was used to assess nodal support. All phylogenetic trees were then visualized using FigTree v1.4.3 (http://tree.bio.ed.ac.uk/software/figtree).

### Data availability.

The RNA sequencing data in this study have been deposited in the NCBI Sequence Read Archive under BioProject accession no. PRJNA631876 and PRJNA626677. All genome sequences of the viruses identified have been uploaded to GenBank under accession no. MT457853 to MT457860.

## Supplementary Material

Supplemental file 1
