# Supplementary material for: Metatranscriptomic Analysis of Virus Diversity in Urban Wild Birds with Paretic Disease
Source: J Virol. 2020 Aug 31;94(18):e00606-20. doi: 10.1128/JVI.00606-20 (PMC7459558; doi:10.1128/JVI.00606-20)
Supplement: Supplemental file 1 [file JVI.00606-20-s0001.pdf]

Supplementary Table 1. Presentation and pathology of Rainbow Lorikeets with Clenched Claw Syndrome

				Degree of Non-suppurative Inflammation					
**Case ID**	**Date**	**Signalment**	**Presentation**	**Cerebrum**	**Brain stem**	**Cerebellum**	**Spinal cord**	**Ganglia**	**Nerve**
U88	19-11-84	J/F	Clenched feet, paresis, ascending neurological signs. Euthanised.	1	2/N1	1	1	-	-
U131	28-10-85	J/-	clenched foot (1), unable to fly, wing tremors, weakness. Died.	0	1/N1	0	2/N2	0	-
U147	28-12-85	J/M	Clenched feet, leg and wing paralysis. Very thin. Moribund.	1	2/N1	2/N1	2/N2	0	0
U150	10-01-86	J/F	Clenched feet. Leg paresis. Wings and head normal.	2	2	2	2/N2	0	-
U205	11-03-86	A/F	Clenched feet, unable to fly, prostrate.	0	0	0	2/N2	2	2/WD
U270	13-05-86	A/M	Leg paralysis. Died.	0	0	0	2/N1	0	WD
U309	25-08-86	A/F	Clenched feet, unable to fly, alert. Euthanasia.	0	0	0	1	0	-
U339	20-10-86	J/M	Clenched feet.	0	2	2	0	0	-
U340	20-10-86	J/F	Clenched feet.	1	1	1	1	0	-
U342	21-10-86	A/M	Clenched feet, eating well, unable to fly or perch.	2	2	2	2/N2	0	WD
U343	21-10-86	A/-	Clenched feet, unable to fly.	0	0	0	2/N1		
U345	25-10-86	A/M	Clenched feet, head tilt, unable to fly or walk. Euthanised. Good condition.	0	2	0	0	0	-
U379	01-12-86	A/M	Clenched feet, unable to fly. Euthanised. Good condition.	0	0/N1	0	0/N1	0	WD
U517	19-06-87	A/M	Clenched feet, progressive neurological signs. Euthanised. Good condition.	0	0	2/N1	2/N1	0	-
U559	10-09-87	J/F	Clenched feet, ataxic, head down. Euthanised. Thin.	1	1	2	1	0	-
U586	05-10-87	J/F	Flew into window. Neurological signs. Euthanised.	0	3	3	2	0	-
U584	28-09-87	A/M	Head bobbing, weak, weak neck. Perching.	0	3	3	3	0	-
U594	21-10-87	J/M	Head nodding. Rolling over. Neurological signs. Euthanasia.	0	2	2	1	0	-
U598	19-10-87	J/M	Neurological signs. Euthanised.	0	2	2	0/WD1	0	-
U645	29-12-97	J/M	Unable to fly then neurological signs. Euthanised.	0	0	2/N1	0	0	-
U654	22-01-88	A/F	Clenched feet, leg paresis. Euthanised Very thin.	0	0	0	2	0	1
U676	05-02-88	-	No history.	0	0	1	1	0	-
U677	18-03-88	J/F	Clenched feet, but able to grip, depressed, opisthotonus, unable to fly. Euthanised. Very thin.	0	2	2	0	0	-
U735	02-06-88	J/M	Neurological signs. Thin. Euthanised.	0	0	0	2	0	1/WD
U782	31-08-88	J/M	Caught be cat. Clenched feet. Leg paresis. Euthanised. Thin	2	0	0	1	1	1
U783	29-08-88	A/M	Clenched feet. Euthanised.	0	0	2/N1	2/N1	0	-
U784	02-09-88	J/F	Clenched feet. Died. Very thin.	1	1	0	2	0	-
U785	19-09-88	A/F	Clenched foot. Unable to fly. Eating well. Euthanised. Good condition.	0	0	0	3	0	2
U793	27-09-88	A/M	Neurological signs. Euthanised. Thin.	0	0	2	2	0	-

U829	13-12-88	J/M	Clenched feet. Euthanised. Good condition.	0	0	2/N1	3	0	-
U831	16-12-88	A/F	Clenched feet. Euthanised. Thin	0	0	0	2/N1	0	WD
U914	17-03-89	A/F	Paresis, unable to use wings. Euthanised. Thin.	0	0	0	2	0	-
U943	21-04-89	A/M	Clenched feet, drooping wing. Euthanised. Good condition.	1	0	0	3	0	1
U1049	12-09-89	J/M	Clenched feet. Very thin.	0	0	2	2	0	-
U1097	1989	-	Clenched feet.	0	0	1	1	0	-
U1107	17-01-90	J/F	Clenched feet. Good condition.	0	0	1	2	2	2
U1123	31-11-90	-	Clenched feet.	-	-	-	2	0	-
U1418	23-11-91	A/F	Paralysis. Euthanasia. Very thin.	0	0	0	2	0	-
U1391	18-12-90	A/F	Clenched feet. Euthanised. Good condition.	0	0	0	3	3	0/WD
U1902	19-01-93	A/M	Clenched foot. Tremor. Euthanised. Thin.	0	0	1/N1	1	2	1/WD
U1903	19-11-93	A/M	Clenched foot. Euthanasia. Good condition.	0	0	0	1/N1	0	1/WD
U2361	01-10-94	A/F	Dog attacked. Head tilt. Lethargic. Euthanised. Thin.	0	3/N2	3/N2	-	-	-
U2408[Q]	10-1994	J/M	Clenched feet. Euthanised.	1	1	1	0	-	-
U2409[Q]	10-1994	-	Clenched feet. Euthanised.	0	2	2	-	-	-
U2463	1995	-	Clenched feet. Weak. Walking on hocks.	0	1	0	3	0	WD
U2464	1995	A/-	Clenched feet. Lateral recumbency.	0	0/WD	0	2	0	WD
U2842[Q]	18-10-96	-	Clenched feet. Euthanised.	0	3	3	3	0	WD
U2945	08-10-97	A/M	Clenched feet. Unable to fly. Euthanised. Good condition.	0	0	0	2	0	-
U2948[Q]	1997	-	Clenched feet. Euthanasia.	0	1	1	2	0	WD
U3099	30-09-98	J/M	Head tilt, ataxia. Euthanised. Very thin.	2	2	2	1	0	-
2989.1	26-07-02	A/F	Clenched feet, head tremor, ataxic. Euthanised. Thin.	0	1	0	-	-	-
3153.1	23-10-02	A/F	Paresis, marked intention tremor. Euthanised. Good condition.	0	1	1	1/WD	0	-
4575.1	01-03-05	A/F	Clenched foot, unable to fly, dyspnoea. Euthanised. Good condition.	0	1	0	-	-	-
5604.1	23-01-07	A/M	Clenched foot, balancing on hocks and wings. Euthanised. Thin.	2	0	0	-	-	-
5789.1	21-05-07	A/F	Depressed, immobile. Euthanised. Emaciated.	0	2	2	-	-	-
**Total tissues with non-suppurative inflammation/total number of tissues examined**				**54**	**54**	**54**	**49**	**47**	**19**
**Total number classified in each grade of severity- 0/1/2/3**				**42/7/5/0**	**28/10/12/4**	**24/9/17/4**	**7/12/23/7**	**41/1/3/1**	

All cases originate from NSW except Queensland cases, which are denoted by [Q].
A - Adult, J - Juvenile, F- Female, M- Male, – - No data.
Non-suppurative inflammation graded on a scale of 0-4 in ascending severity. Necrosis (N) graded on a scale of 0-4 in ascending severity. Wallerian Degeneration (WD) indicated when present.

Supplementary Table 2 Presentation and pathology of passerines with myocardial degeneration and myocarditis.

Species	Case ID	Date	Location	Signalment	Presentation	Myodegeneration/ inflammation	Cardiac myodegeneration/ inflammation	CNS inflammation	Hepatic necrosis/ inflammation	Enteric necrosis/ inflammation	Pancreatic inflammation	Vasculopathy
Magpie	3630.3	1/08/2003	Budgewoi	J/M	Found dead. Good condition. Mass mortality (n=35).	0/0	0/1	0	0/0	0/2	0	1
Currawong	3630.4	1/08/2003	Budgewoi	A/M	Found dead. Good condition. Mass mortality.	0/0	0/1	0	0/0	0/0	0	0
Currawong	3667.1	27/08/2003	Balmoral	A/F	Weak, paresis. Euthanised. Good condition. Mass mortality (n=4).	3/3	1/1	1	0/2	0/0	0	0
Magpie	3687.1	1/08/2003	Canley Heights	A/M	Found dead. Diarrhoea.	0/0	0/1	0	0/0	0/0	0	0
Magpie	5103.1	20/02/2006	Tuggerah	J/M	Paresis, weak, alert, diarrhoea. Euthanised. Thin. Mass mortality (n=23).	0/1	0/1	1	0/0	0/1	0	1
Magpie	5103.2	15/02/2006	Tuggerah	J/M	Paresis, dyspnea. Euthanised. Thin. Mass mortality.	0/0	2/0	1	0/0	0/2	0	1
Magpie	5103.3	16/02/2006	Kulnura	A/F	Recumbent, weak, flaps wings, alert. Euthanised. Good condition. Mass mortality.	0/1	0/1	1	0/0	0/2	0	2
Magpie	5103.4	23/02/2006	Wyong	A/F	Weak, recumbent, alert. Good condition. Mass mortality.	1/2	0/2	2	0/0	0/0	0	1
Magpie	5103.5	12/02/2006	Kincumber	A/M	Recumbent, weakness, flaps wings, alert. Euthanised. Thin. Mass mortality.	1/2	1/1	1	0/0	0/0	0	2
Magpie	5103.6	16/02/2006	Woy Woy	A/F	Weak, aggressive and alert. Euthanised. Thin. Mass mortality.	0/1	2/1	0	0/1	0/0	0	1
Magpie	5103.7	16/02/2006	Umina	A/M	Weak, aggressive and alert. Euthanised. Thin. Mass mortality.	0/2	1/1	0	0/0	0/0	0	1
Magpie	5103.8	14/02/2006	Umina	A/M	Weak. aggressive and alert. Euthanised. Thin. Mass mortality.	0/0	1/1	1	0/0	0/0	0	1
Magpie	5103.9	25/02/2006	Bateau Bay	A/F	Died in Transit. Euthanised. Good condition. Mass mortality.	0/1	2/0	0	0/1	0/0	2	2
Magpie	5103.10	25/02/2006	Bateau Bay	A/M	Paresis, moribund. Euthanised. Thin.	1/1	1/1	1	0/0	0/0	0	1
Magpie	5103.11	24/02/2006	Wyoming	A/M	Weakness. Euthanised. Thin. Mass mortality.	0/1	1/1	1	0/1	0/1	0	0
Raven	5103.12	8/03/2006	Kulnura	J/M	Recumbent, clenched feat, weak, slow righting reflex. Thin. Mass mortality.	0/0	1/0	2	0/0	2/2	0	0
Magpie	5104.1	21/02/2006	Fairlight	J/M	Weakness. Parasites. Euthanised. Very thin.	0/1	0/0	1	0/0	0/1	0	0
Currawong	5109.1	22/02/2006	Kingsford	J/M	Weak, flaps wings well, alert. Euthanised.	0/0	0/1	0	0/0	0/0	0	1
Currawong	5119.1	26/02/2006	Coogee	A/M	Weak, alert. Died. Very thin.	2/1	1/0	0	0/2	0/0	0	0
Magpie	5120.1	27/02/2006	Matraville	A/M	Weak, alert. Died. Thin.	0/1	1/1	1	0/0	0/0	0	1
Magpie Lark	5127.1	8/03/2006	Leichardt	A/F	Weak. Euthanised. Thin.	0/0	2/0	0	0/1	0/0	0	1
Currawong	5134.2	12/03/2006	Kirrawee	A/M	Weak, slow righting, aggressive and alert. Euthanised. Thin.	2/2	0/1	1	0/0	0/0	0	0
Raven	5135.1	14/03/2006	Killarney Height	A/M	Paresis, aggressive, alert. Thin.	1/0	2/0	1	0/0	1/1	0	0
Raven	5168.1	31/03/2006	Balmoral	A/M	Recumbent, weak, dyspnoeic, bloody diarrhoea. Thin.	0/0	0/1	1	2/1	0/1	0	1
Currawong	5519.1	25/11/2006	Kensington	J/-	Weak, ataxia, slow righting. Died. Good condition.	1/1	1/1	0	0/1	1/0	0/1	1
Currawong	5519.2	25/11/2006	Kensington	J/-	Paresis, weakness, poor neck control. Euthanised. Good condition.	-	0/1	0	0/1	0/0	0	1
Currawong	5618.1	24/11/2006	Cromer	A/-	No history. Died. Good condition.	-	0/2	0	2/2	0/0	0	0
Currawong	5618.2	23/11/2006	Cromer	A/-	No history. Died. Good condition.	0/2	1/2	0	2/2	0/0	N/A	0
Currawong	5606.1	23/01/2007	Ashfield	A/M	Found dead. Good condition. Mass.	-	1/1	0	1/1	0/0	0	0

Magpie	5624.1	2/02/2007	Randwick	A/M	Paralysis except head, alert. Died. Good condition.	2/0	0/1	0	1/1	0/0	0	0
Currawong	6736.1	2/02/2009	Manly	A/F	Paresis, flaps wings and eats. Euthanised. Thin.	-	0/1	1	0	0/0	1	0
Currawong	6751.2	12/02/2009	Oyster Bay	A/M	Paresis. Weak. Gurgling. Died. Thin.	1/2	2/2	1	0/1	0/0	1	0
Magpie	6739.1	5/01/2009	Hurstville	J/M	Weak, hock siting, withdrawal reflexes, diarrhoea. Euthanised. Thin.	0/1	0/1	2	0/0	0/0	0	1
Raven	7139.1	21/08/2009	Fairlight	J/-	Weak, ataxic, head tilt, circling. Euthanised. Thin.	0/0	1/0	3	2/0	0/0	0	0
Raven	7200.1	1/10/2009	Balgowlah	J/-	Ataxic, odd head movements, unable to perch, but eating and alert. Euthanised. Thin.	0/0	0/1	2	2/0	0/2	1	2
Currawong	7886.1	22/11/2010	Mosman	J/M	Nestling - fell out of nest. Euthanised. Good condition.	0/0	3/3	0	2/2	0/0	1	2
Currawong	7886.2	22/11/2010	Mosman	J/-	Nestling - fell out of nest. Euthanised. Thin.	0/0	3/3	0	2/2	1/2	2/2	2
Magpie	7912.1	1/12/2010	Balmoral	J/-	Nestling - fell out of nest, parasites. Euthanised. Emaciated.	0/0	0/1	0	0/1	0/2	0	2
Magpie	8536.1a	18/02/2012	Aitkenvale QLD	A/F	Sudden death in rehabilitation care. Good condition.	0/0	0/1	1	N/A	0/0	0	0
Magpie	8536.1b	18/02/2012	Aitkenvale QLD	A/F	Sudden death in rehabilitation care. Good condition.	0/0	0/2	-	0/2	0/0	0	0
Figbird	8599.2	24/04/2012	Haberfeld	A/F	Found dead. Not toxins. Good condition. Mass mortality (n=13).	0/0	0/2	0	0/0	0/2	0	1
Magpie	9585.2	29/10/2013	Nowra	J/M	Fledglings in care, anorexia, lethargy, parasites. Died. Thin. Mass mortality (n=21).	1/2	2/2	1	1/1	2/2	2	2
Magpie	9585.3	29/10/2013	Nowra	J/F	Fledglings in care, anorexia, lethargy, parasites. Died. Thin. Mass mortality.	0/2	0/2	1	0/1	0/2	0	2
Currawong	9586.1	29/10/2013	Mosman	J/M	Nestling - fell out of nest. Euthanasia. Good condition.	0/2	3/2	2	2/0	0/0	1	1
Magpie	9900.1	10/05/2014	Avalon	A/F	Weakness. Not toxins. Good Condition. Mass mortality (n=18).	0/1	0/3	0	0/0	1/2	2	2
Magpie	9900.3	10/05/2014	Avalon	J/M	Weakness. Not toxins. Good Condition. Mass mortality.	0/1	0/1	0	0/0	0/2	0	0
Raven	10444.1	19/02/2015	Mosman	J/F	Weak, recumbent, head curled under body, dyspneoa. Thin.	0/0	1/0	2	0/0	0/2	0	0
Magpie	10592.1	20/05/2015	Oak Flats	A/F	Weak. Mass mortality. Euthanasia. Fenthion detected 3/4 liver samples. Good condition. Mass mortality (n=17).	0/0	0/2	0	2/0	2/2	0	2
Magpie	10592.2	20/05/2015	Oak Flats	A/M	Weak. Mass mortality. Euthanasia. Fenthion detected 3/4 liver samples. Good condition. Mass mortality (n=17).	0/0	0/2	0	2/2	0/2	1	2

A – Adult, J – Juvenile, F- Female, M- Male, - signifies not available for examination.

Myodegeneration, inflammation, necrosis, and vasculopathy graded on a scale of 0-4 in ascending severity.

Locations are within NSW unless otherwise specified. Shaded cases represent clusters or epizootics.

Supplementary Table 3. PCR primers used to amplify viral sequences from bird tissues.

**Primer IDs**	**Forward primer sequence (5'-3')**	**Reverse primer sequence (3'-5')**	**Target**	**Amplicon size (bp)**
Clenched claw syndrome				
BRDV-cap-F/R	CTTGTAGTGGDATCCABCCG	GTGGAGCACCTCTVACTGC	Circovirus-cap	596
MET011/12	AACCAGATCCTCGGTATACC	TGGACCTCTCTTGTGATAGC	Paramyxovirus	301
MET013/14	ATAATGACATGCGATGTGCTC	CTAATGCTAAGATCAAGTCCTACC	Hepatovirus	220
MET150/151	TGCTTAATCCAATTTTTAATCCAGA	CATCCTGGACACATTGTTATTCA	Adenovirus-pol1	113
MET154/155	TGATTTTATTCTGTGATTTCCATG	GGTGACACTGACAGCTTATT	Adenovirus-pol2	78
MET181/182	CTGATAATGGAGCTTATACAACGG	TGTGTATCCAATGCAGTATACGG	Parvovirus	248
Black and white bird diseases				
MET029/030	CCTTTCCTGATCAGTGTTACC	TATTGAGATGGACTGGACTCG	Astrovirus	195
MET009/010	GTTGACGATCACATCAAAGTG	ATTCAGACAGATAGGTATCAACC	Picornavirus	220
MET031/032	TAGATCTGCAGGGTCTAGTG	CCATCCTGATCAAGTCTGG	Polyomavirus	147

Supplementary Table 4. Primers used to recover the full length genomes of the viruses identified here.

**Primer IDs**	**Forward primer sequence (5'-3')**	**Reverse primer sequence (3'-5')**	**Target region**	**Amplicon size (bp)**
Polyoma S1	GGTAGTGACGGTATTTTGAGGTC	CAAGTAGATTCACCTCAGCTCTC	220-1815	1605
Polyoma S2	GCTGCTATAACTGCTTTAGAAGGT	CAGAAAGATAAAGCCCGTCACC	1050-2587	1538
Polyoma S3	GACCCTACTCTTAAAGCCAGACT	TTCTCACGGGTGCCAGTTTAA	2368-3571	1204
Polyoma S4	ATGTGGAATCTCGGTGAAAGTC	TGAAACGCCTTAATTGCCTGA	3223-4968	1736
Polyoma S5	AGTGTGGATCTTGCAGCTTCA	GTCACTATATGCCTGTAAATCGTCC	4896-815	1024
Paramyxo S1	CACAGACTATGATAAGTTGCAGG	GTTGGTTACTGCTTGGATCATG	137-2125	1989
Paramyxo S2	GCAAATCCCAATTCCAGCAAC	CTTCACATGAGGATTGATGGAGG	1396-3343	1948
Paramyxo S3	TGAATGCTGGTCTCAATGAATGG	CTCTTGGAGAAGGGTTTGTTGAC	3272-5308	2037
Paramyxo S4	GAACAGAAGGAAAGAGTGATTACG	AGAGGTCTGGATATTATGGACGA	4953-6653	1701
Paramyxo S5	GAATCATGGAGGAGGTTGACC	GCCCACAATGAGCCTCTAAT	6572-8725	2154
Paramyxo S6	GCACTGTCTAATTCCCCTGATT	GATCTGTGTACATAAGGACCATCT	7988-10547	2560
Paramyxo S7	CGTCTTAAATTCCATTACTGCGC	CAAGTCATGTGGTGGGTTGTAT	9973-12029	2057
Paramyxo S8	GCAACTCATATCTGTGACTTCTTC	GGATCTCAAATGGCAAGTCATG	11546-13613	2068
Paramyxo S9	TCATGGCGTCCGTTATTACAAG	AAGTCTTGGATGAAACCCTTGG	13332-15487	2156
Paramyxo S10	GCACCTATTCATGAGTTGTTGAC	GTTTCACTTCATGTCTAGTCAGAG	14847-16512	1666

Adv_hexon	CACATAGCGGGTCTTTAGCAAC	CAGTTGCAAAAGGAGTTCTGAAG	14550-16376	1827
Adv_pol	TGTCTTCTTTGGTAGCGAAAC	ATTCTTCTTGCGAGCCAGAT	4557-5864	1308
